# Retracted: An ErChen and YinChen Decoction Ameliorates High-Fat-Induced Nonalcoholic Steatohepatitis in Rats by Regulating JNK1 Signaling Pathway

**DOI:** 10.1155/2019/4046908

**Published:** 2019-10-20

**Authors:** Evidence-Based Complementary and Alternative Medicine

*Evidence-Based Complementary and Alternative Medicine* has retracted the article titled “An ErChen and YinChen Decoction Ameliorates High-Fat-Induced Nonalcoholic Steatohepatitis in Rats by Regulating JNK1 Signaling Pathway” [1], because there is image duplication in the article's figures.

In Figure 2, the panels ECYCD and Rosiglitazone show overlapping parts of the same image. In Figure 8, the beta-actin blot in 8(a) is the same as the beta-actin blot in 8(e), flipped vertically, and the beta-actin blot in 8(b) is the same as the beta-actin blot in 8(c), flipped vertically.

We asked the authors to provide all the underlying data and original uncropped images. They provided some of the data and explained that these errors occurred during revision. We confirmed that the beta-actin controls for JNK1 and P-IRS-1sec307 in Figure 8 were flipped vertically at revision. However, we found that while Figure 4 in the original version (equivalent to Figure 2 in the published article) is different to the published figure, panels 4(c) and 4(e) are also from the same field of view, but represent ECYCD and polyenephosphatidylcholine, respectively. The editorial board recommended retraction of the article.
